# Recent Developments in Amber Biomolecular Simulations

**DOI:** 10.1021/acs.jcim.5c01063

**Published:** 2025-07-29

**Authors:** David A. Case, David S. Cerutti, Vinícius Wilian D. Cruzeiro, Thomas A. Darden, Robert E. Duke, Mahdieh Ghazimirsaeed, George M. Giambaşu, Timothy J. Giese, Andreas W. Götz, Julie A. Harris, Koushik Kasavajhala, Tai-Sung Lee, Zhen Li, Charles Lin, Jian Liu, Yinglong Miao, Romelia Salomon-Ferrrer, Jana Shen, Ryan Snyder, Jason Swails, Ross C. Walker, Jinan Wang, Xiongwu Wu, Jinzhe Zeng, Thomas E. Cheatham III, Daniel R. Roe, Adrian Roitberg, Carlos Simmerling, Darrin M. York, Maria C. Nagan, Kenneth M. Merz

**Affiliations:** † Department of Chemistry and Chemical Biology, Rutgers University, Piscataway, New Jersey 08854, United States; ‡ Department of Chemistry, 3463 The University of Florida, 440 Leigh Hall, Gainesville, Florida 32611-7200, United States; § 6857 National Institute of Environmental Health Sciences, 111 TW Alexander Dr., Durham, North Carolina 27709, United States; ∥ Department of Chemistry, University of North Carolina at Chapel Hill, Chapel Hill, North Carolina 27599, United States; ⊥ Advanced Micro Devices Inc., Austin, Texas 78735, United States; # Laboratory for Biomolecular Simulation Research, Institute for Quantitative Biomedicine and Department of Chemistry and Chemical Biology, Rutgers University, Piscataway, New Jersey 08854, United States; ○ San Diego Supercomputer Center, 8784 University of California San Diego, La Jolla, California 92093-0505, United States; □ Department of Pharmaceutical Sciences, 15513 University of Maryland School of Pharmacy, Baltimore, Maryland 21201, United States; △ Laufer Center for Physical and Quantitative Biology, 12301 Stony Brook University, Stony Brook, New York 11794, United States; ▽ Department of Chemistry, Stony Brook University, Stony Brook, New York 11794, United States; ■ Department of Chemistry and Department of Biochemistry and Molecular Biology, 3078 Michigan State University, East Lansing, Michigan 48824-1322, United States; ● Cleveland Clinic, Lerner Research Institute, 9620 Carnegie Avenue, N Building, Cleveland, Ohio 44106, United States; ▲ Cold Start Therapeutics, San Diego, California 92126, United States; ⬡ Beijing National Laboratory for Molecular Sciences, Institute of Theoretical and Computational Chemistry, College of Chemistry and Molecular Engineering, 12465 Peking University, Beijing 100871, China; ⬢ Department of Pharmacology and Computational Medicine Program, University of North Carolina−Chapel Hill, Chapel Hill, North Carolina 27599, United States; ▼ Iambic Therapeutics, 5627 Oberlin Drive, Suite 120, San Diego, California 92121, United States; a Department of Chemistry and Biochemistry, University of California−San Diego, 9500 Gilman Drive, La Jolla, California 92093, United States; b Laboratory of Computational Biology, National Heart, Lung, and Blood Institute (NHLBI), 2511 National Institutes of Health (NIH), Bethesda, Maryland 20892, United States; c Department of Medicinal Chemistry, 7060 The University of Utah, 30 South 2000 East, Salt Lake City, Utah 84112, United States; d Laboratory of Computational Biology, National Heart, Lung, and Blood Institute, National Institutes of Health, Bethesda, Maryland 20892, United States

## Abstract

Amber is a molecular dynamics (MD) software package first conceived by Peter Kollman, his lab and collaborators to simulate biomolecular systems. The *pmemd* module is available as a serial version for central processing units (CPUs), NVIDIA and Advanced Micro Devices (AMD) graphics processing unit (GPU) versions as well as Message Passing Interface (MPI) parallel versions. Advanced capabilities include thermodynamic integration, replica exchange MD and accelerated MD methods. A brief update to the software and recently added capabilities is described in this Application Note.

## Graphical abstract

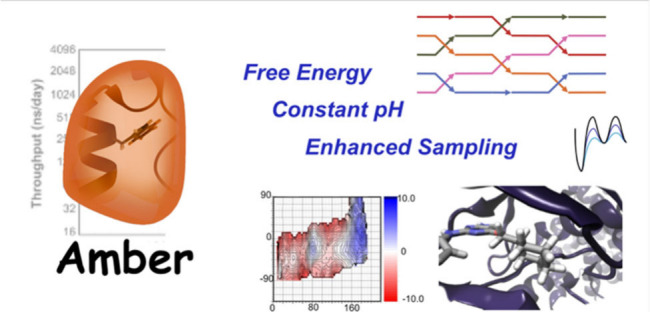

## 1 A Brief History

The Amber biomolecular simulation package began in Peter Kollman’s group about 45 years ago,^1^ and the early history has been summarized elsewhere.^2^ By about 1995, Amber developers had converged on the using the particle-mesh Ewald (PME) model to deal with long-range electrostatic effects,^3,4^ and the *sander* module had become the primary vehicle for molecular dynamics (MD) simulations. *Sander* has a parallel implementation^5,6^ in which forces are distributed among Message Passing Interface (MPI) processes, but the coordinates of all atoms are available to each process at every step. This data structure allows for all parts of the potential energy calculation to be assigned flexibly among processes, but entails additional collective communication. Around 2003, Bob Duke, working with Lee Pedersen and Tom Darden, created a significant new MD engine, called *pmemd*, that distributed both coordinates and forces among processes, introduced dynamic load-balancing, and optimized cache utilization and memory layout. This development continued from Amber versions 8–12, or from about 2003 to 2010.

In 2008, most of the project modules (including *sander*) were split off into an open-source collection called AmberTools,^7^ and *pmemd* was distributed as Amber, which provided in source-code form but with a license that included restrictions on use and redistribution. Both AmberTools and Amber support standard molecular dynamics simulations but AmberTools is required for system setup and analysis. For instance, general triclinic unit cells, including but not limited to rectangular and octahedral boxes, can be used but there is no support for symmetry elements (such as screw axes) that involve rotations. In 2012, *pmemd* was ported to NVIDIA GPUs allowing for significantly accelerated MD capabilities and is what is now distributed as Amber.^8–11^ It has since been extended by multiple groups to support additional, more complex algorithms including implicit solvent models,^10^ replica exchange,^12^ various accelerated MD methods,^13,14^ nudged elastic band (NEB)^15^ and thermodynamic integration (TI)^16–20^ to highlight a few.

This Application Note summarizes additions to *pmemd* in the time period of 2015–2025.

## 2 GPU Accelerated pmemd

Since GPUs are designed to operate as massively parallel computation engines, they are well-suited to the computational demands of MD simulations. By utilizing the highly parallel architecture of GPUs, as well as a leveraging a novel single/fixed precision model (SPFP),^11^ *pmemd* can achieve up to 100-fold speedups compared to traditional central processing unit (CPU)-based simulations and has continued to see performance improvements with each new model of GPU (see Figure 1).

**Figure 1:**
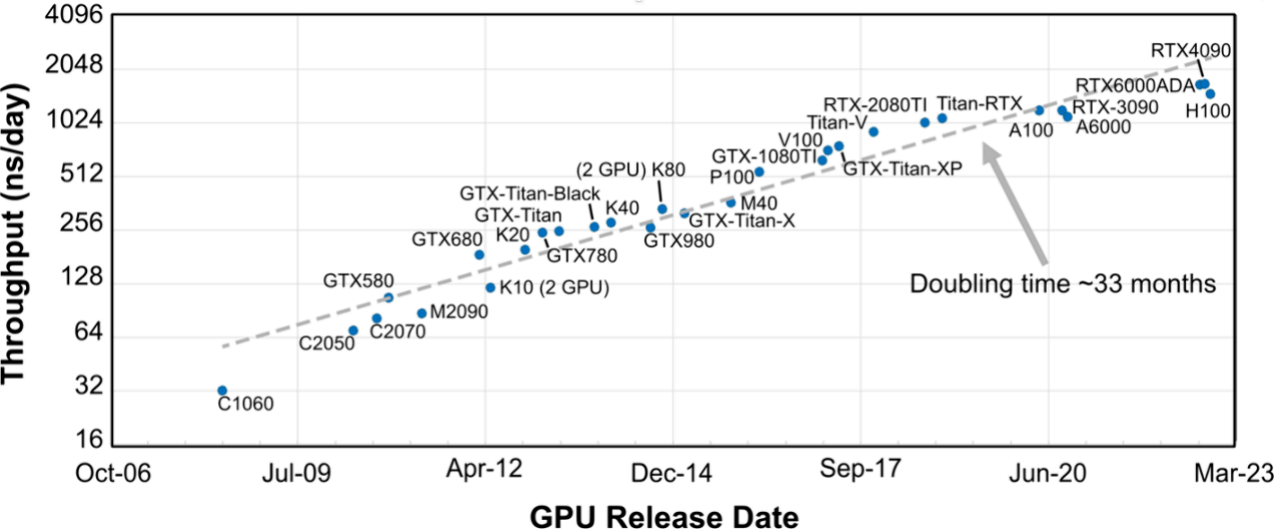
Historical performance of the Amber GPU accelerated *pmemd*. The DHFR 4.0 fs NVE benchmark on different GPU models is shown. All benchmarks are executed on a single GPU.

At the time of writing, the GPU implementation of *pmemd* from Amber 2024 yields performance of about 1.7 μs per day for the 23000 atom DHFR 4 fs NVE benchmark running on a single RTX 4090 GPU, and over 82 ns/day for the 1.07 million atom Satellite Tobacco Mosaic Virus 4 fs benchmark on the same hardware. While performance with smaller systems such as DHFR have leveled out over the past few years, larger systems are still seeing near linear improvement on newer GPUs such as H100, RTX5080 and B200 SXM. As an example, for the DHFR benchmark, a B200 SXM card is no faster than an RTX 4090 card, for but the much larger STMV system, the B200 SXM result of 114 ns/day is 40% faster than the RTX 4090 (albeit for a considerably higher purchase price).

Amber has recently expanded GPU implementations beyond NVIDIA to those manufactured by Advanced Micro Devices (AMD). Execution on AMD GPUs using ROCm and Heterogeneous-Compute Interface for Portability (HIP) are now possible. ROCm is an AMD software stack for GPU programming. HIP is a C++ Runtime API that allows developers to create portable applications for AMD and NVIDIA GPUs. Amber 2024 can be executed on different AMD accelerator architectures including AMD Instinct MI100, MI210, MI250­(X), and MI300A. Timings are updated periodically as new GPU Hardware is released. Along with software dependencies and library compatibility, these are posted when available at the Amber Web site^21^ under the *GPU Support* page.

In summary, the development of the Amber *pmemd* program and its integration with GPU architectures has brought about a revolution in MD simulations by making simulations of biologically relevant time scales possible on cost-effective desktop hardware.

## 3 Neighbor Lists and Nonbonded Interactions on GPUs

Nonbonded interactions in Amber support the common Coulombic and Lennard-Jones nonbonded potentials. Neighbor lists are essential to the efficiency of periodic MD programs, but also impart most of the complexity. If the problem were to compute the interaction of each particle to every other, a basic Ewald sum could be applied to all pairwise interactions, but the problem then has *N^2^* complexity in the number of particles *N*. The neighbor list subdivides the problem into spatial regions or clusters of contiguous atoms, allowing the program to calculate interactions which have a good chance of lying within a cutoff distance of one another. The remainder of the interactions, which are farther apart, are negligible for Lennard-Jones interactions. Coulombic interactions are then handled with the PME algorithm of *N* log­(*N*) complexity. Inextricable from this neighbor list is the notion of whether an interaction is excluded, such as bonded atoms, and on GPUs, the ordering of the particles in memory must also conform to some sort of spatial locality. These compounding requirements have driven the evolution of unique neighbor lists in Amber and other codes.

Amber subdivides the problem into spatial regions, parallelepipeds which span the simulation box, which are at least the length of the cutoff between any two opposing faces. Because there is no check on whether a particular periodic image of the interaction between two particles is correct, the simulations are prepared so that all opposing faces of the simulation cells are separated by at least three cutoff lengths. A Hilbert space-filling curve is inscribed within each spatial region to provide a framework for sorting the atoms inside such that the order of their indices in computer memory follows the physical locality of their coordinates in the simulation. Atoms selected from any contiguous sequence in the list are then likely to be near neighbors of one another. This process is accomplished by a GPU library sorting function. *Tiles* are then produced for the interactions between any two adjacent spatial regions by taking 16 contiguous atoms from one region (*senders*) and checking all atoms of adjacent regions until 32 *receivers* can be found which are within range of at least one of the *senders*. This collection of 32 × 16 tiles is then stored in memory, along with bit masks indicating the exclusion status of any interaction in the tile. The neighbor list is updated once any one particle has migrated far enough to create a potential interaction that has not been accounted within one of the tiles.

## 4 Controlling Solution pH or Redox Potential During MD

There are two different implementations of constant pH simulations in Amber. One employs Metropolis Monte Carlo methods, in which discrete protonation states are sampled and one is based upon lambda values, with partial protonation states (PME-CpHMD). Both methods can be performed in two modes, independent of pH or pH replica exchange.

### 4.1 Metropolis Monte Carlo Constant pH

Amber contains the implementations of the discrete or hybrid MC/MD constant pH method,^22,23^ which utilizes Metropolis Monte Carlo steps for protonation state sampling at specified MD intervals. The discrete constant pH method, which samples only physically meaningful protonation states, can be executed either in GB solvent^22^ or explicit solvent.^23^ One advantage is that multiple residues can be titrated in a single simulation, and each time point corresponds to a physically meaningful binary protonation state of the system (i.e., each titratable group either has a proton or does not). A disadvantage is that the Monte Carlo step to evaluate the protonation change acceptance probability is carried out with an implicit GB solvent energy calculation, even when the underlying simulation uses explicit solvent.

### 4.2 Continuous Constant pH Molecular Dynamics (CpHMD)

CpHMD^24^ propagates fictitious lambda particles (representing the progress of protonation states) alongside real particles (atoms) according to Newton’s equation of motion. The GPU-accelerated particle mesh Ewald (PME)-CpHMD implementation in Amber fully eliminates the dependency of constant pH simulations on implicit GB solvent model.^25^ Forces on both real and lambda particles are calculated in explicit solvent, allowing protonation states to be directly determined by the atomic environment, including ions, lipids, and nucleic acids. Thus, the PME-CpHMD method is particularly suited for studying proton-coupled conformational dynamics of transmembrane proteins and nucleic acids. An asynchronous replica exchange algorithm allows the use of any number of GPUs^26^ for PME-CpHMD REMD. The PME-CpHMD method builds upon the GBNeck2-CpHMD method in Amber,^27,28^ which utilizes GBNeck2 ^29^ for both conformational and protonation state sampling. The GBNeck2-CpHMD method is particularly suited for p*K*_a_ calculations,^28^ including challenging residues such as cysteines^30^ and lysines.^31^

### 4.3 Constant Redox Potential MD

Due to the mathematical similarities between the Henderson–Hasselbalch and Nernst equations, CpHMD methods can be applied to electrochemistry. The only difference is that now a simulation at constant redox potential requires a cycle that contains both reduced and oxidized forms.^32,33^ Discrete constant pH and redox potential MD simulations (C­(pH,E)­MD) can also be carried out. Some additional information is provided in Section 5.4, below.

## 5 Replica Exchange Molecular Dynamics (REMD)

The support for different variations of REMD, an enhanced sampling method,^34,35^ has expanded in recent Amber versions, and a new python tool is included to help users set up the inputs for complex REMD simulations. Examples of useful REMD methods are given in the following subsections. Generally exchange attempts can be made between even/odd partners. Users have the option of implementing a randomly selected pair in Hamiltonian REMD (H-REMD) simulations and or limiting exchange between the first and last replicas.

### 5.1 Constant Pressure (NPT) REMD

Originally limited to exchanging only temperatures in the NVT ensemble, *pmemd* now supports the use of NPT simulations with REMD. When system volume can change, as in an isothermal–isobaric ensemble, the probability *P* of observing a system in a particular configuration is related to pressure and volume in addition to energy.^36^ The specific equations implemented in Amber are shown below:

1 $$P\left(X_{i},E_{a}\right)=\frac{e^{-E_{a}\left(X_{i}\right)\beta_{a}}e^{-\beta_{a}P_{a}V\left(X_{i}\right)}}{Z_{a}}$$

where *P*_*a*_ is the external pressure on the system and *V*(*X*_*i*_) is the volume of the system with coordinates *X*_*i*_. This changes the Hamiltonian replica exchange probability delta to

2 $$\Delta_{H,P}=\left(\beta_{b}\Delta E_{b}-\beta_{a}\Delta E_{a}\right)+\left(\beta_{b}P_{b}-\beta_{a}P_{a}\right)\left(V\left(X_{j}\right)-V\left(X_{i}\right)\right)$$

and the temperature Hamiltonian replica exchange (i.e., when *E*_*a*_ is the same as *E*_*b*_) probability to

3 $$\Delta_{T,P}=\Delta \beta \Delta E+\left(\beta_{b}P_{b}-\beta_{a}P_{a}\right)\left(V\left(X_{j}\right)-V\left(X_{i}\right)\right)$$

The right-most term in the Hamiltonian and temperature deltas can be thought of as a “correction” that can be added to the constant volume deltas:

4 $$\Delta_{\mathrm{correction}}=\left(\beta_{b}P_{b}-\beta_{a}P_{a}\right)\Delta V$$

This PV correction term is added to the Metropolis calculation to account for changes to periodic box dimensions and has been incorporated into Amber when the isothermal–isobaric ensemble is active. Lastly, communication is minimized by the transfer of thermostat temperatures between replicas after a successful exchange, rather than coordinates and velocities. The resulting trajectory files written by an individual MD simulation therefore represent continuous sampling of coordinate space, with changing thermostat temperature. These individual “walker” trajectories can readily be converted to trajectories for each thermostat temperature via postprocessing in *cpptraj*.^37^

### 5.2 H-REMD

Hamiltonian replica exchange is also supported in *pmemd*. Each replica can load a different topology file, allowing alteration of force field parameters between different replicas. Alternately, replicas can vary a parameter in their Amber input files, allowing for many different applications. A few examples include the use of different restraints for umbrella sampling, or different boost strength for accelerated MD. Compared to the traditional approach of using a single MD walker per Hamiltonian, H-REMD allows multiple walkers to contribute to ensemble generation for each Hamiltonian, speeding convergence. In H-REMD, the Metropolis criterion involves evaluation of the energy for each coordinate set using each Hamiltonian. From a practical standpoint, evaluation of the energy for a coordinate set in the alternate Hamiltonian is accomplished by communicating the coordinates to the replica in which that force field or restraint set are already set up. Since this communication is required, the output trajectory files are continuous in Hamiltonian. If desired, conversion to trajectories with continuous sampling of coordinate space is carried out with *cpptraj*. The similarity of the detailed balance equation for H-REMD to that for free energy perturbation (FEP) allows Amber to carry out replica exchange FEP (REFEP) and simultaneously report Δ*G* values between pairs of windows.^38^

### 5.3 pH-REMD

Solution pH can also be varied across different replicas,^39^ allowing for constant pH REMD. This can provide a significant advantage over single-pH simulations, since the titration curves can be strongly dependent on pH. When simulating at a pH far from the p*K*_a_ value, the probability of sampling alternate protonation states can be low, which can lead to kinetic trapping in a conformation that favors the current protonation state. Visiting alternate pH values can facilitate conformational changes that are coupled to pH, speeding convergence of constant pH simulations and predicted p*K*_a_ values. pH-REMD simulations can be carried out in either implicit or explicit solvent.^23^

### 5.4 Redox and Coupled Redox-pH REMD

In Amber, REMD can be carried with replicas sampling different solution pH values, as well as different redox potentials. Using 2D-REMD, both can be varied (E, pH-REMD) for enhanced sampling efficiency when using the discrete protonation state variant of pH-REMD.^40^ A study demonstrated the use of C­(pH,E)­MD to investigate coupled redox and pH effects in a small protein with four heme groups that have distinct redox and pH profiles, but it is very hard to assign particular p*K*_a_/*E*_0_ to individual hemes.^41^

### 5.5 Multidimensional REMD

Amber now supports multidimensional REMD,^12^ where users can define combinations of the above tools. For example, replicas can vary by Hamiltonian in one dimension, and temperature in another. Exchange attempts are carried out between replicas that vary only in one dimension. Since the computational costs grow rapidly, users are cautioned to carefully consider which dimensions are most likely to improve sampling for the specific problem being studied.

### 5.6 Reservoir REMD

A combined Monte Carlo + MD approach using the reservoir REMD method^42,43^ has been implemented in which users can load a set of alternate conformations in a pregenerated structure reservoir. Periodically, exchanges are attempted between the currently sampled conformation and one selected randomly from the reservoir. Reservoir REMD rapidly accelerates the ensemble convergence of REMD simulations. The method has been applied to the simulated folding of peptides/proteins^44^ and RNA,^45^ estimating the impact of mutations,^46^ as well as refinement and reranking of alternate ligand poses^47^ generated using virtual screening.

## 6 Alchemical Free Energy

Alchemical free energy (AFE) methods^48^ leverage artificial “alchemical” pathways to efficiently predict free energy difference between states, and can be applied to gain insight into chemical processes such as the transfer of a molecule from an aqueous to lipid environment, the change in protonation state of a titratable residue in a protein or nucleic acid, or the binding of a drug-like molecule (ligand) to a protein target.

The infrastructure for conducting AFE simulations has been greatly extended in Amber to include a wide range of new methods. A number of new features enable optimization of alchemical transformation pathways.^49,50^ As a start, these include the use of “smoothstep” functions^51^ in the weights used for the Hamiltonian mixing, and new form of softcore potentials^49^ that maintain balance between Coulomb attractions and short-ranged Lennard-Jones repulsions. Users can “soften” the Lennard-Jones and electrostatic interactions with increasing values of α and β parameters, respectively (see ref 49 for details). Further flexibility is provided through lambda-scheduling features that allow customized transformations to be performed. Specifically, different energy terms can be transformed over distinct lambda subintervals.^52^ For example, a traditional stepwise “decharge-vdW-recharge” transformation,^53^ that in previous versions of the code would require 3 separate simulations, could be combined now in a single-step transformation. Second, an alchemical enhanced sampling (ACES) method has been implemented for robust AFE simulations for a wide range of applications.^54^ The ACES method leverages the new optimized alchemical transformation pathways along with Amber’s existing Hamiltonian replica exchange molecular dynamics (H-REMD) framework that has recently been extended for use in the NPT ensemble. Third, new tools for customizing the lambda-spacing through optimization of the phase space overlap lead to improved H-REMD and ACES sampling.^55^ Fourth, scaffold-hopping (core-hopping) relative binding free energy (RBFE) and absolute binding free energy (ABFE) capability are enabled through lambda-dependent Boresch bond, angle, and torsion restraints, and enhanced by lambda-dependent RMSD-fitting restraints to floating reference molecular scaffolds. Fifth, AFE simulations can be conducted using equilibrium thermodynamic integration or free energy perturbation methods, or using a new nonequilibrium work framework and application of Jarzynski^56^ and Crooks^57^ equations. Sixth, end-state free energy corrections using an “indirect” (sometimes referred to as “book-ending”) approach (e.g., MM → QM, MM → MLP, or MM → QM + MLP) are possible for a wide range of generalized hybrid quantum mechanical (QM) and machine learning potentials (MLP).^58–61^ These free energy simulations can be performed using either equilibrium^62^ or nonequilibrium^59^ methods. Seventh, network-wide alchemical free energy analysis of thermodynamic graphs with cycle closure and experimental constraints^63^ is enabled through the latest version of FE-ToolKit.^7,64^ FE-Toolkit is a versatile software suite for the automated analysis of free energy surfaces, minimum free energy paths, and alchemical free energy networks (thermodynamic graphs).^64^ Finally, these methods have been integrated into workflows for production free-energy simulation setup and analysis.^65^

## 7 Improved Sampling in a Single MD Simulation

Both REMD and AFE calculations are parallel processes. If single CPU or GPU sampling is needed, new improved sampling methods are under development in Amber.

### 7.1 Self-Guided Langevin Dynamics

The self-guided (SG) molecular simulation methods, namely, the self-guided molecular dynamics (SGMD)^66,67^ and the self-guided Langevin dynamics (SGLD)^68–73^ were developed for efficient conformational searching and are implemented in Amber. SG methods do not rely on *a priori* energy barrier information to enhance sampling. Instead, they achieve an enhanced conformational search by promoting low frequency motion, which is extracted through a simple local averaging scheme during simulations. SGMD/SGLD has been applied to many studies of long time scale events such as peptide folding,^74–77^ conformational reorganization,^78^ conformational state recognition,^79^ and conformational transitions.^80–82^

### 7.2 GaMD

Gaussian accelerated MD (GaMD) is an established enhanced sampling technique that has been implemented in Amber since 2012.^83,84^ In GaMD calculations, a harmonic boost potential is added to smooth the biomolecular potential energy surface^83^ and reduced the system energy barriers. GaMD accelerates biomolecular simulations by orders of magnitude. When the rare events are not known in advance, the method is advantageous because a predefined reaction coordinate or collective variables are not required. This enables unconstrained sampling of large biomolecular complexes. Since the GaMD boost potential exhibits a Gaussian distribution, biomolecular free energy profiles can be approximately recovered through cumulant expansion to the second order. Moreover, Amber includes novel ligand GaMD (LiGaMD),^85–87^ peptide GaMD (Pep-GaMD),^88^ and protein–protein interaction GaMD (PPI-GaMD),^89^ which allow for binding thermodynamics and kinetics calculations of small molecules, peptides, and proteins, respectively.

## 8 The “Middle” Thermostat

As an alternative thermostat, the middle thermostat scheme also leads to accurate configuration distribution of the constant temperature ensemble (e.g., NVT or NPT ensemble), regardless of whether the thermostat is stochastic or deterministic.^90–92^ For example, it yields a new, more efficient, and robust integrator that achieves accurate joint distribution of volume and configuration for the isobaric–isothermal (constant-NPT) ensemble.^93^ In comparison to conventional MD integrator algorithms, the middle thermostat scheme increases the time step size (i.e., time interval) by a factor of 4–10 for obtaining converged results. The middle thermostat scheme for flexible force fields as well as for force fields with holonomic constraints (e.g., fixed bond length) has been integrated into *pmemd*, *pmemd.MPI*, and *pmemd.cuda*.

## 9 Force Fields

### 9.1 General

The force fields supported in Amber are distributed in AmberTools^7^ annually and are not covered here. However, it is worthwhile to note that each molecule type (e.g., protein, RNA, DNA, ligand, carbohydrate, lipid, etc.) or ion that is incorporated into the users’ system and simulated with *pmemd* has its own force field. Choosing the correct combination is important to simulation reliability and recommendations can be found at the Amber Web site^21^ under the *Force Fields* page.

### 9.2 Integration of 12–6–4 Nonbonded Potentials for Metal Ions

Amber 2024 enhances the flexibility of a previously established 12–6–4 LJ nonbonded model^94^ by allowing the users to manually scale up/down the polarizability of ligand atoms according to several recent parametrization schemes.^95,96^ The new model is hence known as a modified 12–6–4 LJ nonbonded model.

Users may find some preparametrized polarizabilities for common metal–imidazole^95^ and metal–acetate^96^ interactions to fulfill the need of simulating metal binding sites containing aspartates, glutamates and histidines. Moreover, Amber 2024 also supports directly defining *C*_4_ coefficients between specific atom pairs. Users may directly use *tLEaP* to apply the highly flexible, yet chemically meaningful *C*_4_ coefficients to any metal-containing systems.

In previous versions of Amber and AmberTools, a dummy atom type is needed to achieve this atom-pair-specific version of modified 12–6–4 LJ nonbonded model, as mentioned in the earlier work.^97^ With the support of Amber 2024, no dummy atom type is needed, and the atom-pair-specific version of modified 12–6–4 LJ nonbonded model can be applied more precisely and efficiently.^20^

## 10 Tutorials

Tutorials are continually maintained and updated on the Amber Web site^21^ under the *Tutorials* page. At that site, a full list of tutorials and descriptions can be found; examples include how to build different simulation systems, how to parametrize nonstandard parameters, generally creating stable systems and running standard MD, general trajectory analysis, some simple case studies, free energy calculations, chemical reactions and equilibria and helpful tools. Noteworthy new tutorials orient users to the middle thermostat, TI with ACES calculations, including a “Quick Start” FE-ToolKit tutorial on how to analyze free energy simulations, and use of 12–6–4 nonbonded potentials in metal ions.

## Data Availability

Amber is free of charge for noncommercial use. Please see the Amber Web site^21^ for full licensing and distribution information. To download Amber, navigate to the Amber Web site under the *Download Amber* section. Software dependence and build directions can be found in the *Installation* section of the Amber Web site (https://ambermd.org).

## e

D.S.C.: Psivant Therapeutics, 451 D Street Suite 205, Boston, MA 02210, United States

## f

V.W.D.C. Architect Therapeutics, San Diego, CA, United States.

## g

R.E.D.: Independent Contractor, 17949 N Advent Ln., Hauser, ID 83854, United States.

## h

M.G.: Microsoft Inc., Cambridge, MA, 02142, United States.

## i

G.G.: Merck & Co., Inc., Boston, Massachusetts 02115, United States.

## j

K.K.: Schrödinger India Private Limited, #147, Channasandra, RR Nagar, Bangalore, India – 560098.

## k

R.C.W.: RCW Computing, 4653 Carmel Mountain Road Suite 308 - PMB# 620, San Diego, CA 92130, United States.

## l

J.Z.: School of Artificial Intelligence and Data Science, University of Science and Technology of China, Hefei 230026, China.

## References

[1] Weiner P. K., Kollman P. A. (1981). AMBER: Assisted Model Building with Energy Refinement.
A General Program for Modeling Molecules and Their Interactions. J. Comput. Chem..

[2] Pearlman D. A., Case D. A., Caldwell J. W., Ross W. S., Cheatham T. E., DeBolt S., Ferguson D., Seibel G., Kollman P. (1995). AMBER, a Package
of Computer Programs for Applying Molecular Mechanics, Normal Mode
Analysis, Molecular Dynamics and Free Energy Calculations to Simulate
the Structural and Energetic Properties of Molecules. Comput. Phys. Commun..

[3] Sagui C., Darden T. A. (1999). Molecular Dynamics
Simulations of Biomolecules: Long-Range
Electrostatic Effects. Annu. Rev. Biophys. Biomol.
Struct..

[4] Essmann U., Perera L., Berkowitz M. L., Darden T., Lee H., Pedersen L. G. (1995). A Smooth Particle Mesh Ewald Method. J. Chem. Phys..

[5] Vincent J. J., Merz K. M. (1995). A Highly Portable
Parallel Implementation of AMBER4
Using the Message Passing Interface Standard. J. Comput. Chem..

[6] Swanson E., Lybrand T. P. (1995). PVM-AMBER: A Parallel
Implementation of the Amber Molecular
Mechanics Package for Workstation Clusters. J. Comput. Chem..

[7] Case D. A., Aktulga H. M., Belfon K., Cerutti D. S., Cisneros G. A., Cruzeiro V. W. D., Forouzesh N., Giese T. J., Götz A. W., Gohlke H., Izadi S., Kasavajhala K., Kaymak M. C., King E., Kurtzman T., Lee T.-S., Li P., Liu J., Luchko T., Luo R., Manathunga M., Machado M. R., Nguyen H. M., O’Hearn K. A., Onufriev A. V., Pan F., Pantano S., Qi R., Rahnamoun A., Risheh A., Schott-Verdugo S., Shajan A., Swails J., Wang J., Wei H., Wu X., Wu Y., Zhang S., Zhao S., Zhu Q., Cheatham T. E., Roe D. R., Roitberg A., Simmerling C., York D. M., Nagan M. C., Merz K. M. (2023). AmberTools. J. Chem. Inf. Model..

[8] Xu, D. ; Williamson, M. J. ; Walker, R. C. Chapter 1 - Advancements in Molecular Dynamics Simulations of Biomolecules on Graphical Processing Units. In Annual Reports in Computational Chemistry, Wheeler, R. A. , Ed.; Elsevier, 2010; Vol. 6, pp 2–19.

[9] Salomon-Ferrer R., Götz A. W., Poole D., Le Grand S., Walker R. C. (2013). Routine
Microsecond Molecular Dynamics Simulations with AMBERon GPUs. 2. Explicit
Solvent Particle Mesh Ewald. J. Chem. Theory
Comput..

[10] Götz A. W., Williamson M. J., Xu D., Poole D., Le Grand S., Walker R. C. (2012). Routine Microsecond Molecular Dynamics Simulations
with AMBERon GPUs. 1. Generalized Born. J. Chem.
Theory Comput..

[11] Le
Grand S., Götz A. W., Walker R. C. (2013). SPFP: Speed without
Compromise—a Mixed Precision Model for GPU Accelerated Molecular
Dynamics Simulations. Comput. Phys. Commun..

[12] Bergonzo C., Henriksen N. M., Roe D. R., Swails J. M., Roitberg A. E., Cheatham T. E. (2014). Multidimensional Replica Exchange
Molecular Dynamics Yields a Converged Ensemble of an RNA Tetranucleotide. J. Chem. Theory Comput..

[13] Miao Y., Sinko W., Pierce L., Bucher D., Walker R. C., McCammon J. A. (2014). Improved Reweighting
of Accelerated Molecular Dynamics
Simulations for Free Energy Calculation. J.
Chem. Theory Comput..

[14] Pierce L. C. T., Salomon-Ferrer R., Augusto F. de Oliveira C., McCammon J. A., Walker R. C. (2012). Routine
Access to Millisecond Time
Scale Events with Accelerated Molecular Dynamics. J. Chem. Theory Comput..

[15] Bergonzo C., Campbell A. J., Walker R. C., Simmerling C. (2009). A Partial
Nudged Elastic Band Implementation for Use with Large or Explicitly
Solvated Systems. Int. J. Quantum Chem..

[16] Kaus J. W., Pierce L. T., Walker R. C., McCammon J. A. (2013). Improving the Efficiency
of Free Energy Calculations in the Amber Molecular Dynamics Package. J. Chem. Theory Comput..

[17] Lee T.-S., Cerutti D. S., Mermelstein D., Lin C., LeGrand S., Giese T. J., Roitberg A., Case D. A., Walker R. C., York D. M. (2018). GPU-Accelerated Molecular Dynamics
and Free Energy
Methods in Amber18: Performance Enhancements and New Features. J. Chem. Inf. Model..

[18] Mermelstein D. J., Lin C., Nelson G., Kretsch R., McCammon J. A., Walker R. C. (2018). Fast and
Flexible GPU Accelerated Binding Free Energy Calculations within the
Amber Molecular Dynamics Package. J. Comput.
Chem..

[19] Giese T. J., York D. M. (2018). A GPU-Accelerated Parameter Interpolation Thermodynamic
Integration Free Energy Method. J. Chem. Theory
Comput..

[20] Lee T.-S., Hu Y., Sherborne B., Guo Z., York D. M. (2017). Toward Fast and
Accurate Binding Affinity Prediction with pmemdGTI: An Efficient Implementation
of GPU-Accelerated Thermodynamic Integration. J. Chem. Theory Comput..

[21] Amber Website. https://ambermd.org (accessed 6/26/25).

[22] Mongan J., Case D. A., McCammon J. A. (2004). Constant pH Molecular
Dynamics in
Generalized Born Implicit Solvent. J. Comput.
Chem..

[23] Swails J. M., York D. M., Roitberg A. E. (2014). Constant pH Replica Exchange Molecular
Dynamics in Explicit Solvent Using Discrete Protonation States: Implementation,
Testing, and Validation. J. Chem. Theory Comput..

[24] Huang Y., Chen W., Wallace J. A., Shen J. (2016). All-Atom Continuous
Constant pH Molecular Dynamics with Particle Mesh Ewald and Titratable
Water. J. Chem. Theory Comput..

[25] Harris J. A., Liu R., Martins de Oliveira V., Vázquez-Montelongo E. A., Henderson J. A., Shen J. (2022). GPU-Accelerated All-Atom Particle-Mesh
Ewald Continuous Constant pH Molecular Dynamics in Amber. J. Chem. Theory Comput..

[26] Henderson J. A., Verma N., Harris R. C., Liu R., Shen J. (2020). Assessment
of Proton-Coupled Conformational Dynamics of SARS and MERS Coronavirus
Papain-Like Proteases: Implication for Designing Broad-Spectrum Antiviral
Inhibitors. J. Chem. Phys..

[27] Huang Y., Harris R. C., Shen J. (2018). Generalized
Born Based Continuous
Constant pH Molecular Dynamics in Amber: Implementation, Benchmarking
and Analysis. J. Chem. Inf. Model..

[28] Harris R. C., Shen J. (2019). GPU-Accelerated Implementation of Continuous Constant pH Molecular
Dynamics in Amber: pKa Predictions with Single-pH Simulations. J. Chem. Inf. Model..

[29] Nguyen H., Roe D. R., Simmerling C. (2013). Improved Generalized
Born Solvent
Model Parameters for Protein Simulations. J.
Chem. Theory Comput..

[30] Harris R. C., Liu R., Shen J. (2020). Predicting
Reactive Cysteines with Implicit-Solvent-Based
Continuous Constant pH Molecular Dynamics in Amber. J. Chem. Theory Comput..

[31] Liu R., Yue Z., Tsai C.-C., Shen J. (2019). Assessing Lysine and Cysteine Reactivities
for Designing Targeted Covalent Kinase Inhibitors. J. Am. Chem. Soc..

[32] Machuqueiro M., Baptista A. M. (2009). Molecular Dynamics
at Constant pH and Reduction Potential:
Application to Cytochrome C3. J. Am. Chem. Soc..

[33] Cruzeiro V.
W. D., Amaral M. S., Roitberg A. E. (2018). Redox Potential Replica Exchange
Molecular Dynamics at Constant pH in AMBER: Implementation and Validation. J. Chem. Phys..

[34] Hansmann U. H. E. (1997). Parallel
Tempering Algorithm for Conformational Studies of Biological Molecules. Chem. Phys. Lett..

[35] Sugita Y., Okamoto Y. (1999). Replica-Exchange Molecular
Dynamics Method for Protein
Folding. Chem. Phys. Lett..

[36] Okabe T., Kawata M., Okamoto Y., Mikami M. (2001). Replica-Exchange Monte
Carlo Method for the Isobaric-Isothermal Ensemble. Chem. Phys. Lett..

[37] Roe D. R., Cheatham T. E. (2013). PTRAJ and CPPTRAJ: Software for Processing
and Analysis of Molecular Dynamics Trajectory Data. J. Chem. Theory Comput..

[38] Meng Y., Sabri Dashti D., Roitberg A. E. (2011). Computing Alchemical Free Energy
Differences with Hamiltonian Replica Exchange Molecular Dynamics (H-REMD)
Simulations. J. Chem. Theory Comput..

[39] Itoh S. G., Damjanović A., Brooks B. R. (2011). pH Replica-Exchange Method Based
on Discrete Protonation States. Proteins.

[40] Cruzeiro V.
W. D., Roitberg A. E. (2019). Multidimensional
Replica Exchange Simulations for Efficient
Constant pH and Redox Potential Molecular Dynamics. J. Chem. Theory Comput..

[41] Cruzeiro V. W. D., Feliciano G. T., Roitberg A. E. (2020). Exploring Coupled Redox and pH Processes
with a Force-Field-Based Approach: Applications to Five Different
Systems. J. Am. Chem. Soc..

[42] Okur A., Roe D. R., Cui G., Hornak V., Simmerling C. (2007). Improving
Convergence of Replica-Exchange Simulations through Coupling to a
High-Temperature Structure Reservoir. J. Chem.
Theory Comput..

[43] Roitberg A. E., Okur A., Simmerling C. (2007). Coupling of Replica Exchange Simulations
to a Non-Boltzmann Structure Reservoir. J. Phys.
Chem. B.

[44] Kasavajhala K., Lam K., Simmerling C. (2020). Exploring Protocols to Build Reservoirs to Accelerate
Temperature Replica Exchange MD Simulations. J. Chem. Theory Comput..

[45] Lam K., Kasavajhala K., Gunasekera S., Simmerling C. (2022). Accelerating
the Ensemble Convergence of RNA Hairpin Simulations with a Replica
Exchange Structure Reservoir. J. Chem. Theory
Comput..

[46] Kasavajhala K., Simmerling C. (2023). Exploring the Transferability of
Replica Exchange Structure
Reservoirs to Accelerate Generation of Ensembles for Alternate Hamiltonians
or Protein Mutations. J. Chem. Theory Comput..

[47] Alcantara J., Chiu K., Bickel J. D., Rizzo R. C., Simmerling C. (2023). Rapid Rescoring
and Refinement of Ligand-Receptor Complexes Using Replica Exchange
Molecular Dynamics with a Monte Carlo Pose Reservoir. J. Chem. Theory Comput..

[48] York D. M. (2023). Modern
Alchemical Free Energy Methods for Drug Discovery Explained. ACS Phys. Chem. Au.

[49] Tsai H.-C., Lee T.-S., Ganguly A., Giese T. J., Ebert M. C., Labute P., Merz K. M., York D. M. (2023). Amber Free
Energy Tools: A New Framework for the Design of Optimized Alchemical
Transformation Pathways. J. Chem. Theory Comput..

[50] Tsai H.-C., Xu J., Guo Z., Yi Y., Tian C., Que X., Giese T., Lee T.-S., York D. M., Ganguly A., Pan A. (2024). Improvements in Precision of Relative Binding Free Energy Calculations
Afforded by the Alchemical Enhanced Sampling (ACES) Approach. J. Chem. Inf. Model..

[51] Lee T.-S., Lin Z., Allen B. K., Lin C., Radak B. K., Tao Y., Tsai H.-C., Sherman W., York D. M. (2020). Improved Alchemical
Free Energy Calculations with Optimized Smoothstep Softcore Potentials. J. Chem. Theory Comput..

[52] Lee, T.-S. ; Tsai, H.-C. ; Ganguly, A. ; Giese, T. J. ; York, D. M. Chapter 7 - Robust, Efficient and Automated Methods for Accurate Prediction of Protein-Ligand Binding Affinities in Amber Drug Discovery Boost. In Free Energy Methods in Drug Discovery: Current State and Future Directions, Armacost, K. A. , Thompson, D. C. , Eds.; ACS Publications, 2021; Vol. 1397, pp 161–204.

[53] Tsai H.-C., Tao Y., Lee T.-S., Merz K. M., York D. M. (2020). Validation
of Free Energy Methods in Amber. J. Chem. Inf.
Model..

[54] Lee T.-S., Tsai H.-C., Ganguly A., York D. M. (2023). ACES: Optimized
Alchemically Enhanced Sampling. J. Chem. Theory
Comput..

[55] Zhang S., Giese T. J., Lee T.-S., York D. M. (2024). Alchemical
Enhanced
Sampling with Optimized Phase Space Overlap. J. Chem. Theory Comput..

[56] Jarzynski C. (1997). Nonequilibrium
Equality for Free Energy Differences. Phys.
Rev. Lett..

[57] Crooks G. E. (1998). Nonequilibrium
Measurements of Free Energy Differences for Microscopically Reversible
Markovian Systems. J. Stat. Phys..

[58] Giese T. J., Zeng J., Lerew L., McCarthy E., Tao Y., Ekesan Ş., York D. M. (2024). Software Infrastructure for Next-Generation
QM/MM-ΔMLP Force Fields. J. Phys. Chem.
B.

[59] Tao Y., Giese T. J., York D. M. (2024). Electronic and Nuclear Quantum Effects
on Proton Transfer Reactions of Guanine-Thymine (G-T) Mispairs Using
Combined Quantum Mechanical/Molecular Mechanical and Machine Learning
Potentials. Molecules.

[60] Zeng J., Tao Y., Giese T. J., York D. M. (2023). Qdπ: A Quantum Deep Potential
Interaction Model for Drug Discovery. J. Chem.
Theory Comput..

[61] Zeng J., Tao Y., Giese T. J., York D. M. (2023). Modern Semiempirical Electronic Structure
Methods and Machine Learning Potentials for Drug Discovery: Conformers,
Tautomers, and Protonation States. J. Chem.
Phys..

[62] Giese T. J., York D. M. (2019). Development of a
Robust Indirect Approach for MM →
QM Free Energy Calculations That Combines Force-Matched Reference
Potential and Bennett’s Acceptance Ratio Methods. J. Chem. Theory Comput..

[63] Giese T. J., York D. M. (2021). Variational Method
for Networkwide Analysis of Relative
Ligand Binding Free Energies with Loop Closure and Experimental Constraints. J. Chem. Theory Comput..

[64] Giese T. J., Snyder R., Piskulich Z., Barletta G. P., Zhang S., McCarthy E., Ekesan Ş., York D. M. (2025). FE-Toolkit: A Versatile
Software Suite for Analysis of High-Dimensional Free Energy Surfaces
and Alchemical Free Energy Networks. J. Chem.
Inf. Model..

[65] Ganguly A., Tsai H.-C., Fernández-Pendás M., Lee T.-S., Giese T. J., York D. M. (2022). Amber Drug Discovery
Boost Tools: Automated Workflow for Production Free-Energy Simulation
Setup and Analysis (ProFESSA). J. Chem. Inf.
Model..

[66] Wu X., Wang S. (1998). Self-Guided Molecular Dynamics Simulation for Efficient
Conformational
Search. J. Phys. Chem. B.

[67] Wu X., Wang S. (1999). Enhancing Systematic
Motion in Molecular Dynamics Simulation. J.
Chem. Phys..

[68] Wu X., Brooks B. R. (2003). Self-Guided Langevin Dynamics Simulation Method. Chem. Phys. Lett..

[69] Wu X., Brooks B. R. (2011). Toward Canonical
Ensemble Distribution from Self-Guided
Langevin Dynamics Simulation. J. Chem. Phys..

[70] Wu X., Damjanovic A., Brooks B. R. (2012). Efficient and Unbiased Sampling of
Biomolecular Systems in the Canonical Ensemble: A Review of Self-Guided
Langevin Dynamics. Adv. Chem. Phys..

[71] Wu X., Brooks B. R., Vanden-Eijnden E. (2016). Self-Guided
Langevin Dynamics via
Generalized Langevin Equation. J. Comput. Chem..

[72] Wu X., Brooks B. R. (2020). Reformulation of
the Self-Guided Molecular Simulation
Method. J. Chem. Phys..

[73] Wu X., Brooks B. R. (2025). Self-Guided Molecular
Simulation to Enhance Concerted
Motion. Int. J. Mol. Sci..

[74] Wu X. W., Sung S. S. (1999). Simulation of Peptide
Folding with Explicit Water—A
Mean Solvation Method. Proteins.

[75] Wu X., Wang S. (2000). Folding Studies of
a Linear Pentamer Peptide Adopting a Reverse Turn
Conformation in Aqueous Solution through Molecular Dynamics Simulation. J. Phys. Chem. B.

[76] Wu X., Wang S. (2001). Helix Folding of an
Alanine-Based Peptide in Explicit Water. J.
Phys. Chem. B.

[77] Wu X., Wang S., Brooks B. R. (2002). Direct
Observation of the Folding
and Unfolding of a β-Hairpin in Explicit Water through Computer
Simulation. J. Am. Chem. Soc..

[78] Damjanović A., Wu X., García-Moreno E. B., Brooks B. R. (2008). Backbone Relaxation
Coupled to the Ionization of Internal Groups in Proteins: A Self-Guided
Langevin Dynamics Study. Biophys. J..

[79] Damjanović A., Miller B. T., Wenaus T. J., Maksimović P., García-Moreno E. B., Brooks B. R. (2008). Open Science Grid
Study of the Coupling between Conformation and Water Content in the
Interior of a Protein. J. Chem. Inf. Model..

[80] Ramans-Harborough S., Kalverda A. P., Manfield I. W., Thompson G. S., Kieffer M., Uzunova V., Quareshy M., Prusinska J. M., Roychoudhry S., Hayashi K. I., Napier R., Genio C. D., Kepinski S. (2023). Intrinsic Disorder and Conformational Coexistence in
Auxin Coreceptors. Proc. Natl. Acad. Sci. U.S.A..

[81] Pendse P. Y., Brooks B. R., Klauda J. B. (2010). Probing the Periplasmic-Open State
of Lactose Permease in Response to Sugar Binding and Proton Translocation. J. Mol. Biol..

[82] Damjanović A., García-Moreno E. B., Brooks B. R. (2009). Self-Guided Langevin
Dynamics Study of Regulatory Interactions in Ntrc. Proteins.

[83] Miao Y., Feher V. A., McCammon J. A. (2015). Gaussian
Accelerated Molecular Dynamics:
Unconstrained Enhanced Sampling and Free Energy Calculation. J. Chem. Theory Comput..

[84] Wang J., Arantes P. R., Bhattarai A., Hsu R. V., Pawnikar S., Huang Y.-m. M., Palermo G., Miao Y. (2021). Gaussian Accelerated
Molecular Dynamics: Principles and Applications. WIREs Comput. Mol. Sci..

[85] Miao Y., Bhattarai A., Wang J. (2020). Ligand Gaussian Accelerated Molecular
Dynamics (LiGaMD): Characterization of Ligand Binding Thermodynamics
and Kinetics. J. Chem. Theory Comput..

[86] Wang J., Miao Y. (2023). Ligand Gaussian Accelerated Molecular Dynamics 2 (LiGaMD2): Improved
Calculations of Ligand Binding Thermodynamics and Kinetics with Closed
Protein Pocket. J. Chem. Theory Comput..

[87] Wang J., Miao Y. (2024). Ligand Gaussian Accelerated Molecular Dynamics 3 (LiGaMD3): Improved
Calculations of Binding Thermodynamics and Kinetics of Both Small
Molecules and Flexible Peptides. J. Chem. Theory
Comput..

[88] Wang J., Miao Y. (2020). Peptide Gaussian Accelerated Molecular
Dynamics (Pep-GaMD): Enhanced
Sampling and Free Energy and Kinetics Calculations of Peptide Binding. J. Chem. Phys..

[89] Wang J., Miao Y. (2022). Protein-Protein Interaction-Gaussian Accelerated Molecular Dynamics
(PPI-GaMD): Characterization of Protein Binding Thermodynamics and
Kinetics. J. Chem. Theory Comput..

[90] Zhang Z., Liu X., Chen Z., Zheng H., Yan K., Liu J. (2017). A Unified
Thermostat Scheme for Efficient Configurational Sampling for Classical/Quantum
Canonical Ensembles Via Molecular Dynamics. J. Chem. Phys..

[91] Zhang Z., Yan K., Liu X., Liu J. (2018). A Leap-Frog Algorithm-Based Efficient
Unified Thermostat Scheme for Molecular Dynamics. Chin. Sci. Bull..

[92] Zhang Z., Liu X., Yan K., Tuckerman M. E., Liu J. (2019). Unified Efficient Thermostat
Scheme for the Canonical Ensemble with Holonomic or Isokinetic Constraints
via Molecular Dynamics. J. Phys. Chem. A.

[93] Liang W., Wang S., Wang C., Wang W., She X., Wang C., Shao J., Liu J. (2025). An Efficient Integrator
Scheme for Sampling the (Quantum) Isobaric-Isothermal Ensemble in
(Path Integral) Molecular Dynamics Simulations. J. Chem. Theory Comput..

[94] Li P., Merz K. M. (2014). Taking into Account the Ion-Induced
Dipole Interaction in the Nonbonded Model of Ions. J. Chem. Theory Comput..

[95] Li Z., Song L. F., Sharma G., Koca Fındık B., Merz K. M. (2023). Accurate Metal-Imidazole Interactions. J. Chem.
Theory Comput..

[96] Jafari M., Li Z., Song L. F., Sagresti L., Brancato G., Merz K. M. (2024). Thermodynamics
of Metal-Acetate Interactions. J. Phys. Chem.
B.

[97] Koca
Fındık B., Jafari M., Song L. F., Li Z., Aviyente V., Merz K. M. (2024). Binding of Phosphate
Species to Ca2+ and Mg2+ in Aqueous Solution. J. Chem. Theory Comput..

